# Calcium phosphate ceramics combined with rhBMP6 within autologous blood coagulum promote posterolateral lumbar fusion in sheep

**DOI:** 10.1038/s41598-023-48878-9

**Published:** 2023-12-12

**Authors:** Natalia Ivanjko, Nikola Stokovic, Marko Pecin, Drazen Vnuk, Ana Smajlovic, Niko Ivkic, Hrvoje Capak, Ana Javor, Zoran Vrbanac, Drazen Maticic, Slobodan Vukicevic

**Affiliations:** 1 Laboratory for Mineralized Tissues, Center for Translational and Clinical Research, School of Medicine, University of Zagreb, Salata 11, 10000 Zagreb, Croatia; 2 Center of Excellence for Reproductive and Regenerative Medicine, School of Medicine, University of Zagreb, Zagreb, Croatia; 3 Clinics for Surgery, Orthopedics and Ophthalmology, Faculty of Veterinary Medicine, University of Zagreb, Zagreb, Croatia; 4 Department of Radiology, Ultrasound Diagnostics and Physical Therapy, Faculty of Veterinary Medicine, University of Zagreb, Zagreb, Croatia

**Keywords:** Medical research, Drug development, Experimental models of disease, Preclinical research, Translational research

## Abstract

Posterolateral spinal fusion (PLF) is a procedure used for the treatment of degenerative spine disease. In this study we evaluated Osteogrow-C, a novel osteoinductive device comprised of recombinant human Bone morphogenetic protein 6 (rhBMP6) dispersed in autologous blood coagulum with synthetic ceramic particles, in the sheep PLF model. Osteogrow-C implants containing 74–420 or 1000–1700 µm ceramic particles (TCP/HA 80/20) were implanted between L4–L5 transverse processes in sheep (*Ovis Aries*, Merinolaandschaf breed). In the first experiment (n = 9 sheep; rhBMP6 dose 800 µg) the follow-up period was 27 weeks while in the second experiment (n = 12 sheep; rhBMP6 dose 500 µg) spinal fusion was assessed by in vivo CT after 9 weeks and at the end of the experiment after 14 (n = 6 sheep) and 40 (n = 6 sheep) weeks. Methods of evaluation included microCT, histological analyses and biomechanical testing. Osteogrow-C implants containing both 74–420 and 1000–1700 µm ceramic particles induced radiographic solid fusion 9 weeks following implantation. *Ex-vivo* microCT and histological analyses revealed complete osseointegration of newly formed bone with adjacent transverse processes. Biomechanical testing confirmed that fusion between transverse processes was complete and successful. Osteogrow-C implants induced spinal fusion in sheep PLF model and therefore represent a novel therapeutic solution for patients with degenerative disc disease.

## Introduction

Osteogrow, an autologous bone graft substitute (ABGS) comprised of recombinant human Bone morphogenetic protein 6 (rhBMP6) within autologous blood coagulum (ABC) as a BMP carrier is a novel osteoinductive device which might be used as a therapeutic option for the treatment of bone segmental defects and for achieving spinal fusion^1^. Osteogrow was proven efficacious and safe in various preclinical models including anterior lumbar interbody fusion (ALIF) in sheep^2^ and posterolateral spinal fusion (PLF) in rabbits and sheep^2, 3^. Moreover, the safety and efficacy of Osteogrow were further tested in clinical trials in patients with distal radial fracture (DRF)^4^ and patients undergoing high tibial osteotomy (HTO) procedure^5^.

However, in certain indications where compressive forces are present, a compression-resistant matrix (CRM) should be added to Osteogrow implants to enhance their biomechanical properties^3, 6^. First, we combined Osteogrow with allograft particles (Osteogrow-A) and tested it in rabbit and sheep PLF model^2, 3^. Following successful outcomes in preclinical testing, Osteogrow-A was evaluated in a double-blinded controlled Phase II study in patients undergoing posterolateral and interbody lumbar fusion (EudraCT number 2017-000860-14). However, allograft use is related to several disadvantages including risk of viral transmission, immunogenicity and regulatory issues in different markets^7, 8^. Moreover, allograft bone is rapidly resorbed thus not representing a good carrier resisting compression.

Synthetic calcium phosphate (CaP) ceramics including tricalcium phosphate (TCP) and hydroxyapatite (HA) are an alternative CRM which might substitute the allograft use. CaP ceramics might be formulated into blocks or particles of different shapes and sizes ranging from 74 µm to 5 mm. Moreover, the resorbability of CaP ceramics might be adjusted by combining highly resorbable TCP with relatively stable HA in various ratios forming biphasic calcium phosphate (BCP)^9^. We have shown that Osteogrow combined with different CaP ceramics (Osteogrow-C) promote new bone formation in a rat subcutaneous bone formation assay^6, 10–12^ and subsequently tested promising formulations in rabbit PLF model^13, 14^. Conducted experiments in rabbits demonstrated that Osteogrow-C promotes bone induction and integration with native transverse processes. However, the rabbit PLF model is an intermediate step in the evaluation of novel bone regenerative therapies with limited translatability to humans due to differences in the spine anatomy, different BMP doses and prolonged time to induce bone formation in higher-order species including humans^15^. On the other hand, the sheep PLF model is a well-established animal model used in the advanced evaluation of novel osteoinductive therapies^2, 16, 17^. The size and distances between sheep lumbar vertebrae are comparable to humans making the sheep PLF model highly translatable to clinics^15^. Therefore, several promising BMP-based osteoinductive strategies, including delivery of BMPs on CaP ceramics or collageneous carrier, have been evaluated in preclinical studies on sheep PLF model^16, 17^. However, till now, none of them eventually resulted in a device approved for clinical use.

In the present study, we aimed to evaluate the efficacy of Osteogrow-C containing biphasic calcium phosphate ceramic particles in promoting posterolateral spinal fusion in the sheep PLF model as a final step of preclinical studies. Moreover, since particle size is among essential properties of ceramics, we aimed to determine whether the size of ceramic particles affects the spinal fusion outcome in sheep. Therefore, we conducted a direct comparison of two Osteogrow-C formulations which differed in the size of ceramic particles (74–420 µm and 1000–1700 µm) and that had previously demonstrated optimal results in rat ectopic bone formation and in the rabbit PLF model^10, 14^.

## Results

### Spinal fusion success rate

**Figure 1:**
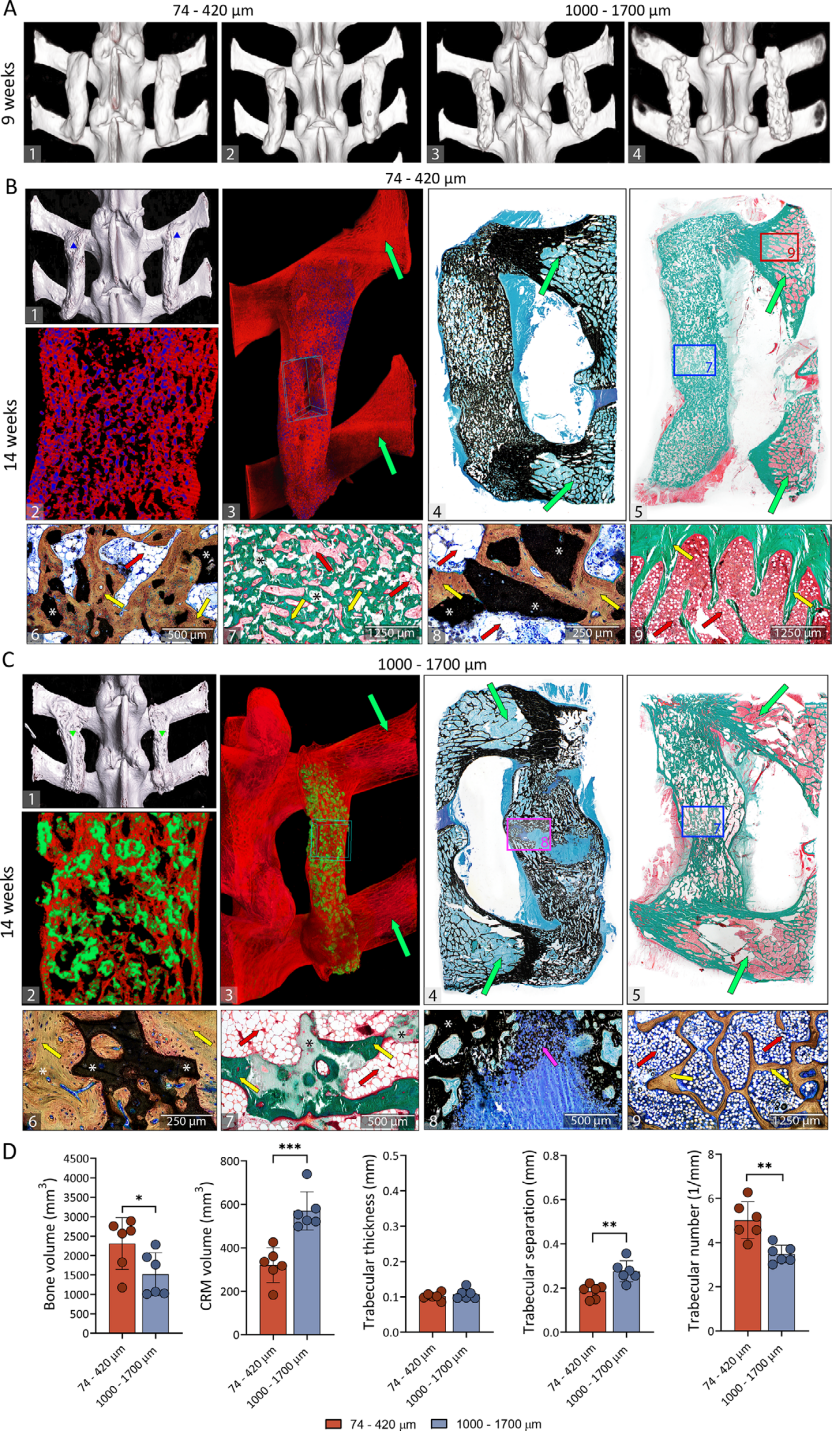
Spinal fusion outcome at 9 and 14 weeks following implantation. (**A**(1–4)) In vivo CT 3D reconstructions of spinal fusion between adjacent transverse processes induced by Osteogrow-C implants containing 74–420 µm (**A**1-2) or 1000–1700 µm (**A**3-4) ceramic particles 9 weeks following implantation. (**B-C**) MicroCT reconstruction and histological features of spinal fusion induced by Osteogrow-C implants containing (**B**) 74–420 µm or (**C**) 1000–1700 µm ceramic particles as observed on week 14 after surgery. The fused spinal segment was reconstructed (**B**1, **C**1) and newly formed bone (red) and small (74–420 µm; blue) (**B**(2–3)) or medium (1000–1700 µm; green) (**C**(2–3)) ceramics were separated on microCT sections. Histological sections revealed that bone induced by Osteogrow-C with 74–420 µm (**B**(4–5)) and 1000–1700 µm (**C**(4–5)) ceramic particles was integrated with adjacent transverse processes. The areas marked with squares in the figures (**B**5,**C**4 and **C**5) are shown enlarged in the figures (**B**7,**B**9,**C**7,**C**8), respectively. Bone was present on the surfaces and between ceramic particles (**B**(6–8) and **C**(6–7)). Ongoing endochondral ossification (pink arrow) was still present in small areas in animals treated with Osteogrow-C with 1000–1700 µm (**C8**). Sections were stained by Goldner (**B**(5,7,9), **C**(5, 7)), Von Kossa (**B**4, **C**(4,8)) or Sanderson’s Rapid Bone Stain with Van Gieson picrofuchsin (**B**(6,8), **C**(6, 9)). Green arrows indicate transverse processes; yellow arrows indicate newly formed bone and red arrows indicate adipocytic bone marrow. The asterisks indicate synthetic ceramics. Scale bars are indicated in the lower left corner. (**D**) Bone volume, CRM volume, trabecular thickness, trabecular separation and trabecular number of induced bone between transverse processes 14 weeks following implantation as determined by microCT analyses. Number of specimens used in microCT analyses was 6 per group. All P values below 0.05 were considered significant and are marked with asterisks: * (p ≤ 0.05), ** (p ≤ 0.01), *** (p ≤ 0.001).

The success of spinal fusion was determined on microCT/CT sections and by palpatory testing of segmental mobility. In the first experiment, analyses of microCT sections revealed that after 27 weeks spinal fusion was achieved in all specimens (9/9) in the group containing Osteogrow-C implants with 74–420 µm ceramic particles and in 88,8% of specimens (8/9) in the group containing Osteogrow-C implants with 1000–1700 µm ceramic particles. All tested vertebral segments in both experimental groups were immobile as determined by palpatory testing. In the second experiment, analyses of CT sections acquired 9 weeks following surgery revealed that radiographic solid fusion was achieved in all specimens (12/12) from both experimental groups (Osteogrow-C containing 74–420 µm and 1000–1700 µm ceramics) (Fig. 1A). Spinal fusion was confirmed by microCT analyses and palpatory testing on weeks 14 and 40 after surgery when the experiment was terminated.

### MicroCT analyses

**Figure 2:**
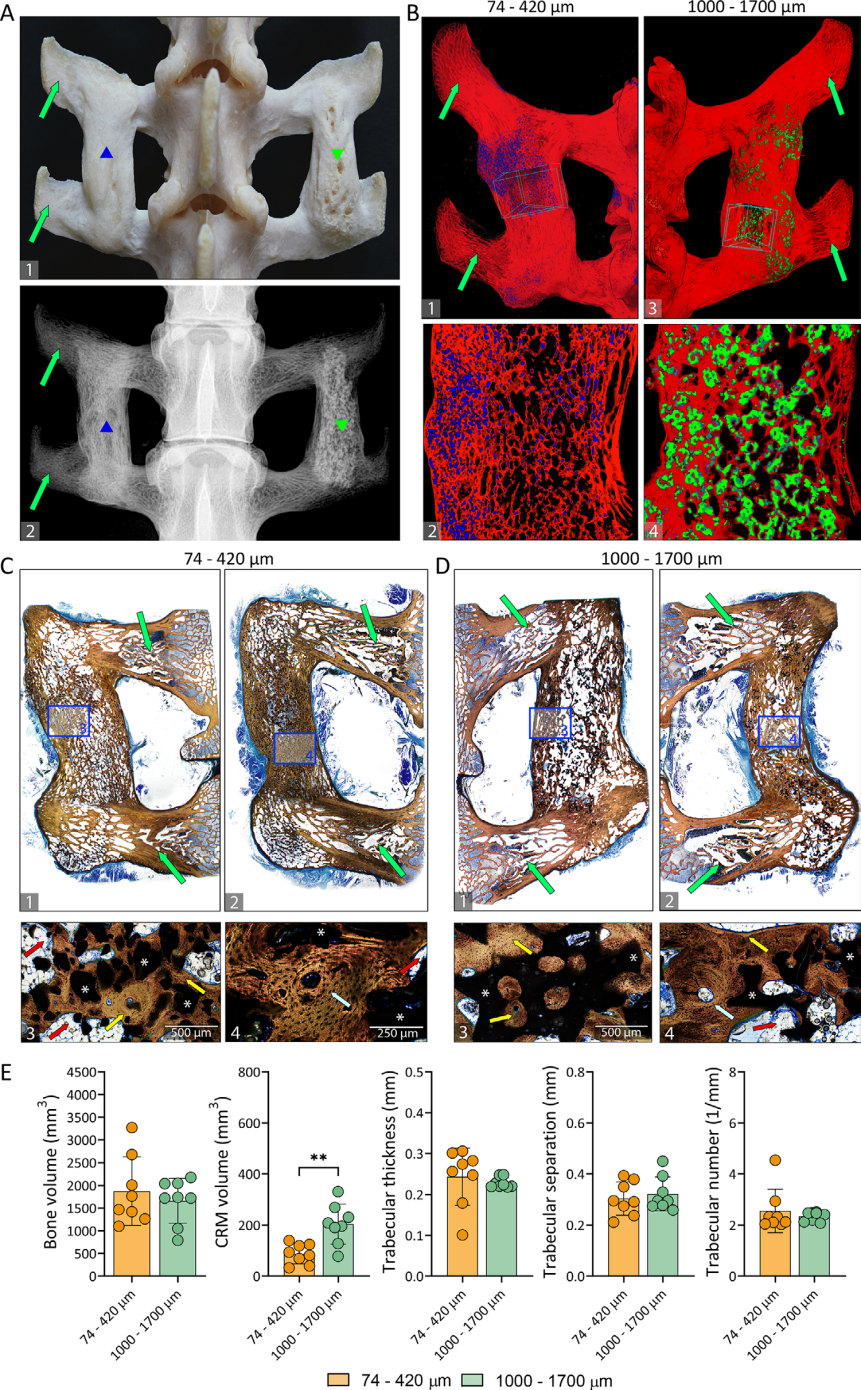
Spinal fusion outcome 27 weeks following implantation. Fused lumbar spinal segments as observed on the (**A**1) macerated specimen, (**A**2) X-ray image, (**B**) microCT and (**C-D**) histological sections. Newly formed bone (red) and ceramic particles in two different sizes ((74–420 µm; blue) (**B**(1–2)); (1000–1700 µm; green)) (**B**(3–4)) were separated on microCT 3D reconstructions and sections. Smaller particles (74–420 µm) (blue) were significantly more resorbed than larger particles (1000–1700 µm) (green). (**C-D**) Histological analyses revealed osseointegration of bone induced by Osteogrow-C with (**C**) 74–420 µm and (**D**) 1000–1700 µm ceramic particles with transverse processes on week 27. The areas marked with squares in the figures **C**(1–2) and **D**(1–2) are shown enlarged in the figures **C**(3–4), and **D**(3–4), respectively. The bone between transverse processes contained both areas of compact and trabecular bone. Osteons (light blue arrow) containing concentrically arranged lamellae forming osteons as well as circumferential lamellae parallel to the surfaces were present in compact bone areas (**C**4, **D**4). Sections were stained by Sanderson’s Rapid Bone Stain with Van Gieson picrofuchsin (**C**,**D**). Green arrows indicate transverse processes; yellow arrows indicate newly formed bone and red arrows indicate adipocytic bone marrow. The asterisks indicate synthetic ceramics. Scale bars are indicated in the lower left corner. (**E**) Bone volume, CRM volume, trabecular thickness, trabecular separation and trabecular number of newly formed bone between transverse processes 27 weeks after surgery as determined by microCT analyses. Number of specimens used in microCT analyses was 9 per group. All P values below 0.05 were considered significant and are marked with asterisks: * (p ≤ 0.05), ** (p ≤ 0.01), *** (p ≤ 0.001).

**Figure 3:**
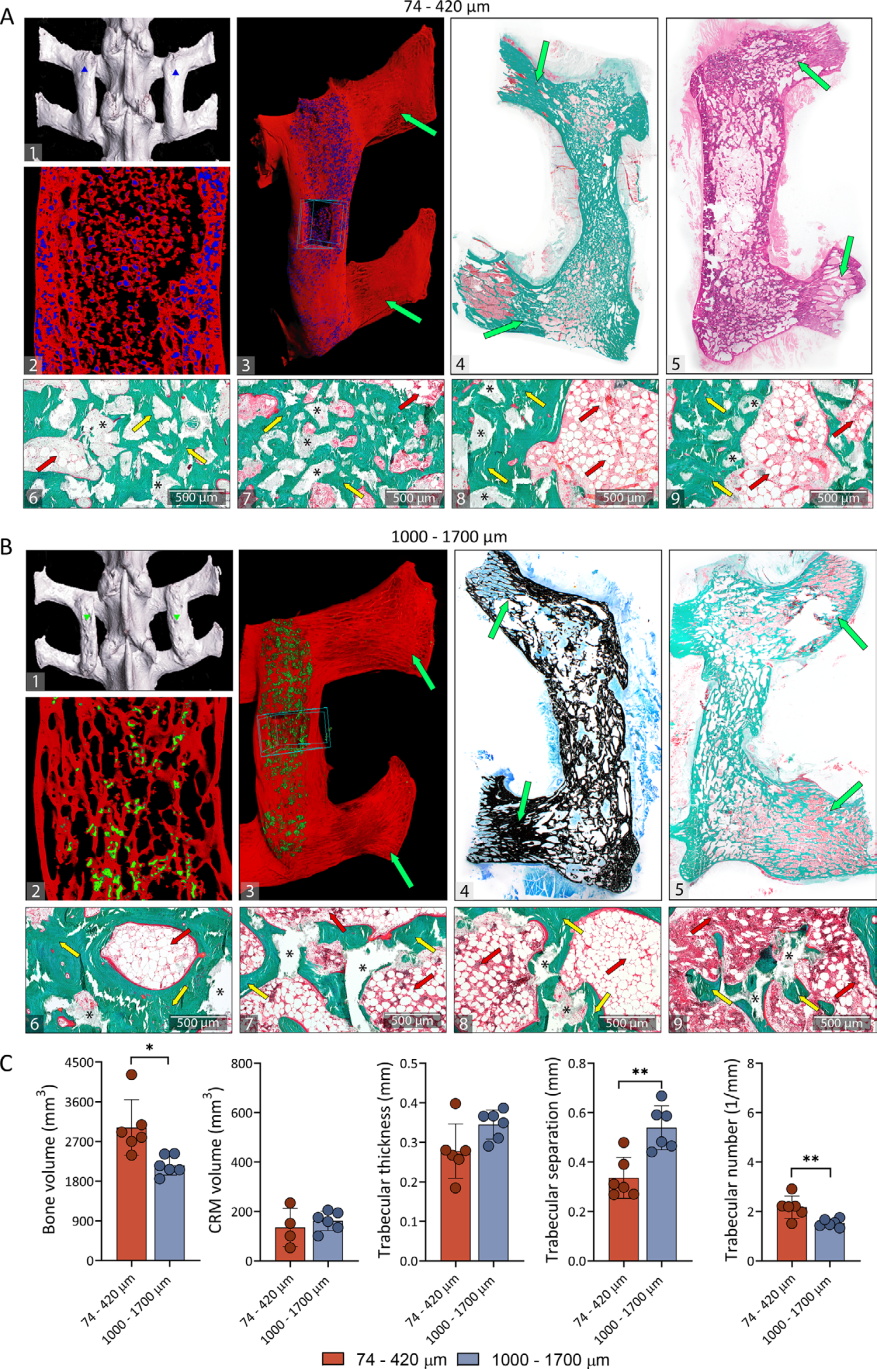
Spinal fusion outcome 40 weeks following implantation. Spinal fusion induced by Osteogrow-C implants with 74–420 µm (**A**) and 1000–1700 µm (**B**) ceramic particles was maintained 40 weeks following surgery as observed on microCT (**A**(1–3), **B**(1–3)) and histological sections (**A**(4–9), **B**(4–9)). Newly formed bone (red) and ceramic particles in two different sizes ((74–420 µm;blue) (**A**(2–3)); (1000–1700 µm; green)) (**B**(2–3)) were separated on microCT 3D reconstructions and sections. Histological analyses revealed that bone containing remnants of 74–420 µm (A(4–5)) and 1000–1700 µm (B(4–5)) ceramic particles formed continuity with native transverse processes. Sections were stained by Goldner (**A**(4,6–9), **B**(5–9)), hematoxylin–eosin (**A**5) or Von Kossa (**B**4) stain. Green arrows indicate transverse processes; yellow arrows indicate newly formed bone and red arrows indicate adipocytic bone marrow. The asterisks indicate synthetic ceramics. Scale bars are indicated in the lower left corner. (**C**) Bone volume, CRM volume and trabecular parameters of bone between transverse processes 40 weeks after surgery as determined by microCT analyses. Number of specimens used in microCT analyses was 6 per group. All P values below 0.05 were considered significant and are marked with asterisks: * (p ≤ 0.05), ** (p ≤ 0.01), *** (p ≤ 0.001).

**Figure 4:**
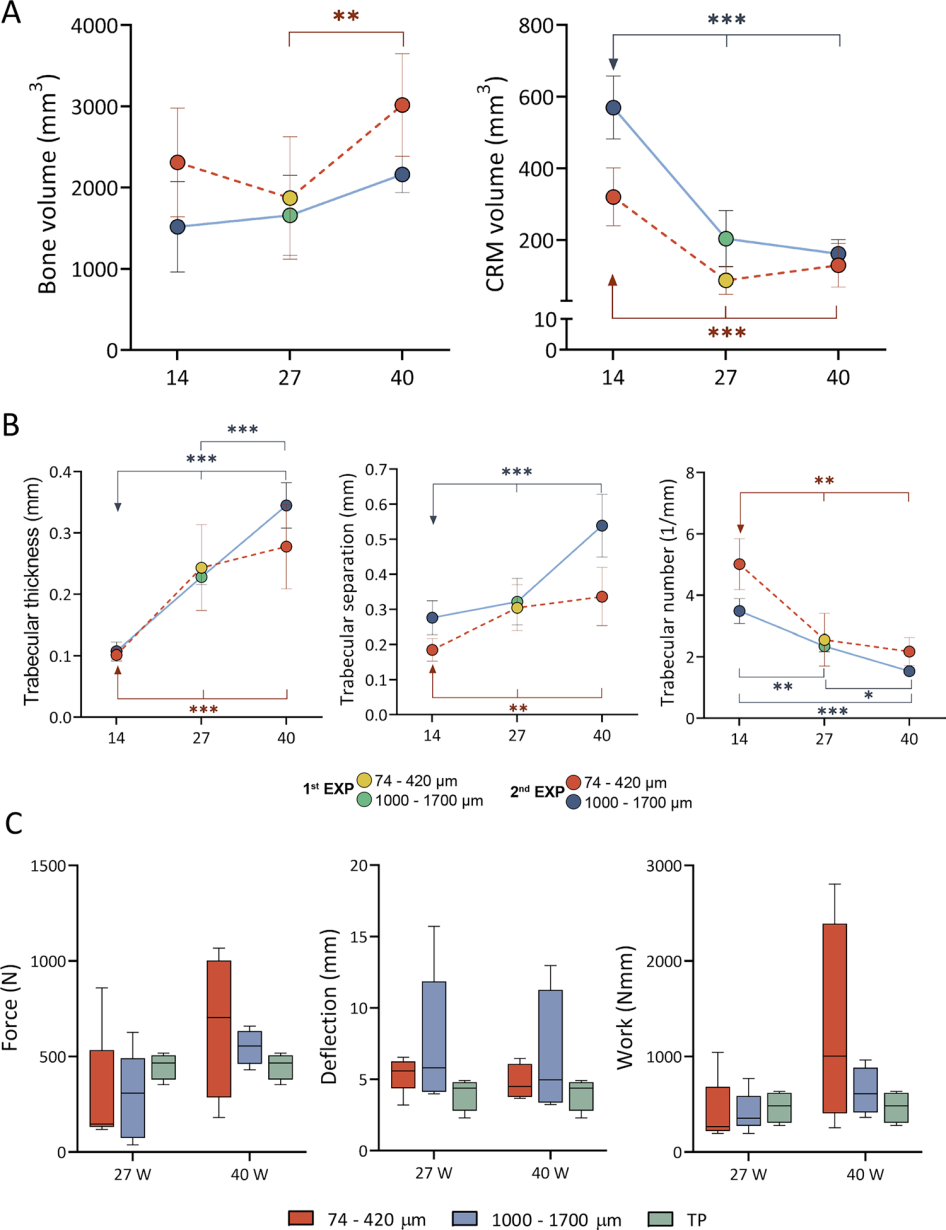
MicroCT and biomechanical parameters through the follow-up period. (**A,B**) Summarized values for bone and CRM volume as well as trabecular parameters induced by Osteogrow-C in two conducted experiments; **Experiment 1** (Osteogrow-C with 74–420 µm (yellow) and 1000–1700 µm (green) particles with 27 week follow-up) and **Experiment 2** (Osteogrow-C with 74–420 µm (orange) and 1000–1700 µm (blue) particles with 14 and 40 week follow-up). Number of specimens used in microCT analyses was 6 (week 14 and 40) or 9 (week 27) per group (**C**). Biomechanical properties (force, deflection and work-to-break) of bone between adjacent transverse processes induced by Osteogrow-C with 74–420 µm (orange) and 1000–1700 µm (blue) ceramic particles on week 27 and 40 after surgery. Number of analysed specimens in three-point bending test was 5 or 4 per group. Transverse processes (TP, green) were used as a control group. All P values below 0.05 were considered significant and are marked with asterisks: * (p ≤ 0.05), ** (p ≤ 0.01), *** (p ≤ 0.001).

Osteogrow-C implants containing 74–420 µm or 1000–1700 µm synthetic ceramic particles in both experiments induced the formation of bone which achieved complete osseointegration with adjacent transverse processes (Figs. 1A,B(1–3),C(1–3), 2A(1,2),B, 3A(1–3),B(1–3)). Bone and ceramic particles were separated on obtained microCT sections and quantified employing microCT analyses (Figs. 1D, 2E, 3C, and 4A,B).

On week 14 following implantation, the volume of newly induced bone was extensive in all Osteogrow-C implants containing ceramic particles and there was no difference among groups containing particles of different sizes (Fig. 1D). Moreover, on the later time points (weeks 27 and 40), bone volume was further increased compared to week 14 (Figs. 1D, 2E, 3C and 4A). Furthermore, trabecular thickness and trabecular separation increased in time while trabecular number decreased in time from week 14 to week 40 (Fig. 4B). Importantly, on the first (14 weeks) and last (40 weeks) time point, trabecular number was higher while trabecular separation was lower in implants containing 74–420 µm particles compared to implants containing 1000–1700 µm particles (Figs. 1D and 3C).

To achieve the desired consistency and distribution of ceramic particles within ABC, Osteogrow-C (74–420 µm) implants were formulated with 2.4 g (1st experiment) or 3 g (2nd experiment) of ceramics, while Osteogrow-C (1000–1700 µm) implants were formulated with 1.6 g (1st and 2nd experiment) of ceramics. However, ceramics volume was significantly lower 14 weeks after implantation in specimens containing 74–420 µm particles compared to specimens containing 1000–1700 µm particles indicating significantly higher resorbability of smaller particles (Fig. 1D). Moreover, on prolonged time points (after 27 and 40 weeks) (Figs. 2E and 3C) ceramics volume was further decreased in both experimental groups and there was present only a limited amount of residual ceramic particles (Fig. 4A).

### Histology

Histological analyses revealed that bone induced by Osteogrow-C implants containing 74–420 µm and 1000–1700 µm ceramic particles was completely integrated with native transverse processes providing solid fusion in specimens obtained from all observed time points (14, 27, and 40 weeks) (Figs. 1B(4–5),C(4–5), 2C(1–2),D(1–2), 3A(4–5),B(4–5)).

On week 14 following surgery newly formed bone between adjacent transverse processes was predominantly lamellar but areas of woven bone were still present. Importantly, newly formed bone contained both compact and trabecular bone (Figs. 1B(6–8),C(6,7)). Compact bone areas contained osteons and a dense bone network between the ceramic particles. Moreover, newly formed bone was also present on the surfaces of ceramic particles as well as in the pores of larger (1000–1700 µm) particles (Fig. 1C(6,7)). Bone surfaces were lined with osteoblasts laying down osteoid and gradually becoming embedded in the bone matrix as well as osteoclasts taking part in bone remodeling. Importantly, BMP-induced osteoclastic activity was not observed in native bone of transverse processes (Fig. 1B(9),C(9)). Small areas of ceramic surfaces did not contain bone and these areas were typically lined with multinuclear giant cells (Fig. 1B(8)). Moreover, mononuclear and multinuclear inflammatory cells were present in the vicinity of ceramic surfaces. Areas of trabecular bone contained trabeculae connecting adjacent ceramic particles. Trabeculae were surrounded by bone marrow containing hematopoietic cells, adipocytes, and blood vessels. Compared to the bone marrow in the native transverse process (Fig. 1B(9),C(9)), bone marrow in newly formed bone was hypocellular and contained less hematopoietic cells but more adipocytes. Importantly, 14 weeks after surgery there were still small areas of ongoing endochondral ossification (Fig. 1C(8)).

At later time points (weeks 27 and 40) the fusion was maintained and newly formed bone was completely integrated with native transverse processes (Figs. 2C(1–2),D(1–2), 3A(4–5),B(4–5)). Bone fusing two adjacent transverse processes was lamellar and, similar to week 14, newly formed bone contained both areas of compact and trabecular bone between ceramic particles. Areas of compact bone contained numerous concentrically arranged lamellae forming osteons (Figs. 2C(4),D(4)) and circumferential lamellae parallel to the surfaces. On weeks 27 and 40, ceramic particles were partially resorbed and the resorption rate was dependent on the particle size since smaller (74–420 µm) particles appeared more resorbed than larger (1000–1700 µm) particles.

### Biomechanical testing

Three-point bending test confirmed that Osteogrow-C implants containing 74–420 µm and 1000–1700 µm particles induced successful and biomechanically competent fusion between adjacent transverse processes 27 and 40 weeks following implantation (Fig. 4C).

At these time points, the maximum force, deflection and work-to-break of specimens from both experimental groups were comparable with transverse processes that served as a control group. The break in the three-point bending test eventually mainly occurred in the central portion of the fusion mass, in between two adjacent transverse processes. Importantly, there was no significant difference in the biomechanical properties among specimens containing 74–420 µm and 1000–1700 µm. However, it should be noted that in both experimental groups maximum force and work-to-break increased in time from week 27 to week 40 while deflection was comparable among these time points (Fig. 4C).

### Pathology dissection

Thorough *post-mortem* examination of animals and pathohistological analyses revealed physiological organs, absence of any abnormality and pathological changes on vital organs as well as absence of any adverse effects related to Osteogrow-C implants including heterotopic ossification.

## Discussion

Degenerative disc disease (DDD) is among the most common and most challenging conditions in clinical practice. Relief of pain related to DDD might be achieved by inducing fusion between affected vertebrae. Spinal fusion might be achieved between adjacent vertebral bodies (anterior, transforaminal and posterior spinal fusion) and between transverse processes (posterolateral spinal fusion). Currently, the gold standard for achieving spinal fusion is autologous bone graft (ABG) which is related to several disadvantages including pain and deformity at the donor site and a limited amount of available bone^17–20^. Therefore, BMP-based osteoinductive strategies have been proposed as an ABG alternative and evaluated in preclinical trials. In these experiments, different BMP carriers including natural polymers^21–25^, synthetic polymers^26–28^, inorganic materials^16, 29–40^ and their combinanation^11, 20, 41–47^ have been evaluated in various animal models in rabbits, dogs, sheep and NHPs. However, till now none of them has been approved for clinical use in PLF indication^15^. We have previously developed a novel osteoinductive device containing rhBMP6 in autologous blood coagulum with synthetic ceramics (Osteogrow-C) and tested it in rabbit PLF model^1, 3, 13, 48^. In the present study, we have demonstrated for the first time that Osteogrow-C is efficient in the sheep PLF model and induces spinal fusion in large animals for an extended period. Moreover, in this study, we have addressed unresolved issues regarding the size of ceramic particles and rhBMP6 dose in implants.

BMP-mediated ectopic bone induction is a relatively rapid process in small rodents (mice, rats) while in larger animals it is significantly slower^49^. Therefore, the follow-up period in sheep studies is typically longer than in smaller animals and successful spinal fusions were reported 12–27 weeks following surgery ^2, 16, 17, 50^. In this study, we have demonstrated that rhBMP6 in ABC with synthetic ceramics induced spinal fusion after 14 weeks as observed by microCT and histology as well as radiographic fusion after only 9 weeks. Relatively rapid bone induction might be attributed to superior osteoinductive properties of rhBMP6, ABC permeability to cell ingrowth and reduced immune response when compared to the use of bovine collagen^1, 3, 51^. We have previously evaluated Osteogrow-C in rabbit PLF study and determined the time course of BMP-mediated bone induction using several time points (7, 14 and 27 weeks following surgical procedure)^13, 14^. Therefore, the completion of the present sheep PLF study allowed us for the first time to compare the time course of BMP-induced osteogenesis between these two species. Importantly, the sequence of events in BMP-induced osteogenesis including changes in radiological, histological, and biomechanical parameters was similar in rabbits and sheep. However, the process was significantly slower in sheep since histological findings on the first endpoint of the present study (14 weeks) resembled findings observed 7 weeks after implantation in rabbits.

The size of ceramic particles used as a scaffold is an important implant determinant but only a few animal studies were focused on the comparison between different sizes of ceramic particles^52^. Some of the previous studies on sheep PLF model used relatively large (2000–4000 µm) ceramic particles for BMP delivery^16^. However, we have previously demonstrated that small ceramic particles (74–420 µm) induce a higher amount of bone than large particles (1000–4000 µm) in rat subcutaneous model^10^. In our rabbit PLF study, we have shown that both small (74–420 µm) and medium (500–1700 µm) particles combined with rhBMP6/ABC might promote spinal fusion^14^. In this study, we have shown that implants with both 74–420 µm and 1000–1700 µm particles induced fusion in sheep and resulted in similar outcomes. Therefore, both of them might be potentially used in the final product. Importantly, implants with smaller particles require a higher amount of ceramics to achieve uniform particle distribution in the implant and therefore in this study implants with 74–420 µm particles contained higher ceramic mass than those with 1000–1700 µm particles (2.4–3 g compared to 1.6 g). However, once the implants were prepared, implants with small (74–420 µm) particles possessed better-handling properties compared to implants with larger particles (> 1000 µm). It should be noted that the present study did not provide a definite verdict on whether particle size affects the biomechanical properties of newly formed bone due to the study limitations including a relatively small number of specimens used in biomechanical testing because a certain number of specimens were histologically processed to present the entire fusion mass including the adjacent transverse processes. Moreover, the applied biomechanical method (three-point bending test) evaluated specimens in only one loading direction, and thus the biomechanical testing was consequently not conducted in all spine loading directions.

In both experiments in this study, we used relatively small (500–800 µg) BMP6 doses compared to other sheep and non-human primates PLF studies in which typical BMP2 doses ranged between 1 and 10 mg^16, 20, 29, 41^. Superior osteoinductive properties of BMP6 compared to BMP2 might be attributed to BMP6 resistance to noggin inhibition^53^. This finding is especially important since reported side effects in previous use of BMP2-containing device including ectopic bone formation, osteolysis, swelling of adjacent tissue and radiculitis resulted from the use of large and supraphysiological doses of BMP2 in these devices^1^. Moreover, it has been previously shown that ABC promotes tight rhBMP6 binding with plasma proteins in the fibrin meshwork and allows a sustained rhBMP6 release^51^. Therefore, the aforementioned side effects might be avoided with the use of this novel osteoinductive device efficacious with a relatively small rhBMP6 dose (500 µg) as demonstrated in this study.

In this study, we have not evaluated how the chemical composition of the ceramics affects the outcome of spinal fusion. However, we have previously compared hydroxyapatite, tricalcium phosphate and biphasic (TCP/HA) calcium phosphate in rat subcutaneous assay and rabbit PLF model^10, 13, 14^ and based on the obtained results selected BCP with an 80/20 ratio to be used in this study. Interestingly, previous studies on sheep PLF model also favored biphasic ceramics containing high TCP proportion or pure TCP^16, 17^. The ratio of TCP to HA determines the resorbability of ceramics since HA is more stable than TCP. As expected, due to the high proportion of TCP, the ceramics used in this study were significantly resorbed till the end of the experiment. Moreover, we have shown that particles affect the resorbability since 74–420 µm particles were significantly more resorbed than 1000–1700 µm particles.

We have previously demonstrated that rhBMP6 in autologous blood coagulum with bone allograft as CRM (Osteogrow-A) promotes spinal fusion in sheep PLF model^2^. However, a comparison of the present results with the outcome of Osteogrow-A studies revealed that synthetic calcium phosphate ceramics are superior CRM to allograft. Namely, Osteogrow-C had a higher fusion success rate, it was efficacious at lower doses and induced more rapid fusion. Therefore, Osteogrow-C is a promising bone regenerative therapy for different skeletal indications^54^ including degenerative spine diseases that remains to be evaluated in clinical trials.

## Conclusions

Osteogrow-C induced relatively rapid, bilateral spinal fusion in sheep PLF model as observed by radiological and histological analyses as well as biomechanical testing. Therefore, Osteogrow-C represents a novel therapeutic solution for achieving spinal fusion in the treatment of patients with degenerative disease of the spine. Moreover, two different sizes of ceramic particles used as a part of Osteogrow-C achieved comparable spinal fusion outcomes providing two efficient Osteogrow-C formulations for application in future phases of Osteogrow-C development.

## Material and methods

### Experimental design

**Figure 5:**
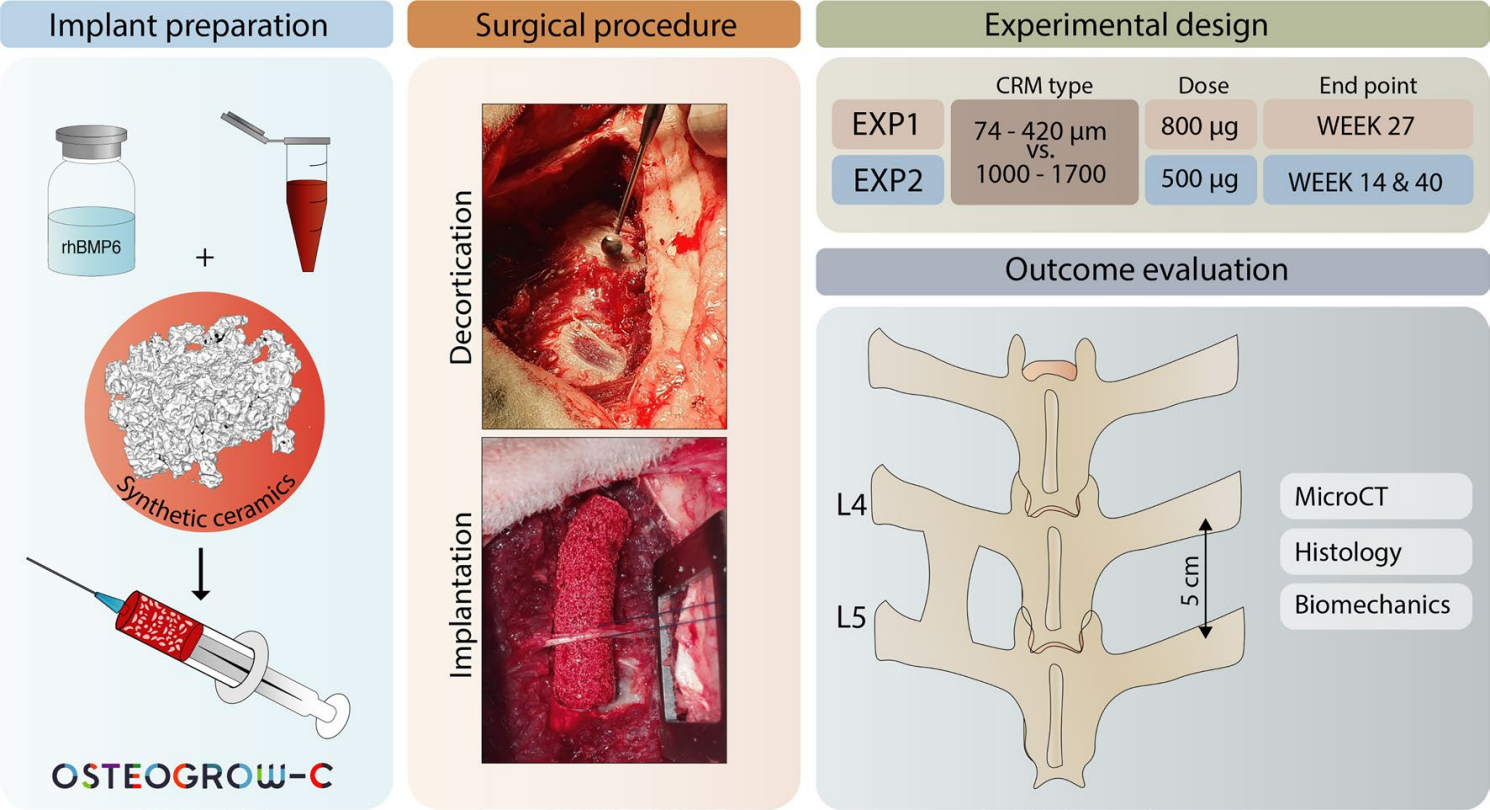
Implant preparation, surgical procedure and experimental design. Osteogrow-C implants were prepared as follows: rhBMP6 was dissolved in water and added to autologous blood withdrawn from sheep jugular vein. Blood was mixed with biphasic calcium phosphate ceramic particles in two different sizes (74–420 µm or 1000–1700 µm) and left to coagulate. Following decortication, Osteogrow-C implants were bilaterally placed between sheep L4–L5 lumbar transverse processes. At the end of observation period spinal fusion outcome was evaluated by microCT, histology, and biomechanics.

In this study, we have conducted two experiments in which we observed spinal fusion outcomes following implantation of Osteogrow-C (rhBMP6/ABC) with two synthetic ceramic particles which differed in the size of ceramic particles (74–420 µm and 1000–1700 µm) (Fig. 5).

In the first experiment nine sheep (*Ovis Aries*, Merinolaandschaf breed, aged 4–5 years) were assigned to the following two experimental groups: (A) rhBMP6/ABC + biphasic calcium phosphate ceramics (BCP, TCP/HA ratio 80/20) 74–420 µm; and (B) rhBMP6/ABC + biphasic calcium phosphate ceramics (BCP, TCP/HA ratio 80/20) 1000–1700 µm. BMP dose (800 µg, 100 µg/mL) and blood volume (800 µL) were the same in all implants while the mass of ceramics was higher in the group with 74–420 µm than 1000–1700 µm particles (2.4 g and 1.6 g respectively) since higher mass was needed to achieve uniform particle distribution. The number of samples was 9 per group and different implants were used to achieve fusion on the left and right side of each animal to minimize biological variability. The follow-up period for all animals was 27 weeks.

In the second experiment twelve sheep (*Ovis Aries*, Merinolaandschaf breed, aged 4–5 years) were assigned to the same experimental groups as in the first experiment: (A) rhBMP6/ABC + biphasic calcium phosphate ceramics (BCP, TCP/HA ratio 80/20) 74–420 µm; and (B) rhBMP6/ABC + biphasic calcium phosphate ceramics (BCP, TCP/HA ratio 80/20) 1000–1700 µm. However, to optimize the Osteogrow-C implants, the applied BMP6 dose was decreased to 500 µg per implant (62.5 µg/mL) while the amount of 74–420 µm ceramic particles was increased to 3 g. The number of samples was 12 per group and the same implant formulation was implanted on the left and right side of each animal. Spinal fusion outcome was assessed 9 weeks after implantation by in vivo CT scans while the animals were killed after 14 (six animals) and 40 weeks (six animals) for further fusion assessment by ex vivo microCT, histological and biomechanical analyses.

Recombinant human BMP6 used in this study was produced by Genera Research (Zagreb, Croatia)^51^ while the evaluated synthetic ceramics were produced by CaP Biomaterials (East Troy, WI, USA)^10^.

### Osteogrow-C implant preparation

Osteogrow-C implants were prepared according to our standard procedure^14^ while rhBMP6 dose and ABC/CRM volumes were optimized for application in sheep PLF model (Fig. 5). In brief, lyophilized rhBMP6 was dissolved in water for injection and drawn into 1 mL syringes while sterilized synthetic ceramic particles were placed into 10 mL syringes. Autologous blood (8 mL) was withdrawn from the sheep jugular vein into empty 10 mL syringes that were subsequently connected by a luer connector with syringes containing rhBMP6. A solution containing rhBMP6 was rapidly mixed with blood and blood containing rhBMP6 was transferred to a 10 mL syringe with synthetic ceramic particles. Ceramics and blood were gently mixed in a syringe until blood coagulation to achieve a homogenous distribution of particles through the implant.

### Surgical procedure and clinical monitoring

Surgical procedures were conducted at the Clinics for Surgery, Orthopedics and Ophthalmology at the Faculty of Veterinary Medicine, University of Zagreb according to the established surgical procedure^2^. Animals were sedated using a combination of xylazine (0.1 mg/kg) and ketamine (5 mg/kg) applied intramuscularly. An intravenous cannula was placed in v.cephalica antebrachii and diazepam (0.2 mg/kg) and thiopental (5–10 mg/kg) were administered intravenously. Intraoperative analgesia was performed with continuous administration of fentanyl (0.1–0.5 µg/kg/min) intravenously with prior administration of a fentanyl bolus (2–5 µg/kg i.v.) Anesthesia was maintained by inhalation of a mixture of oxygen and isoflurane. The sheep was placed in a sternal position and a skin incision was made in the median line in the area of the fourth and fifth lumbar vertebrae (L4–L5), followed by a fascial paramedian incision. Transverse processes of the L4–L5 were accessed between the muscles, *m.multifidus* and *m.longissimus*. The decortication of the transverse processes was done using a high-speed drill (35,000 RPM) and previously prepared Osteogrow-C implants were placed bilaterally between the transverse processes (Fig. 5). Subsequently, the muscles, fascia, subcutaneous tissue, and skin were reconstructed using standard techniques.

Anesthesia protocols and postoperative analgesia for animals used in experiment were well prepared before the start of the surgical procedures. Postoperative analgesia was maintained with meloxicam (0.5 mg/kg, SID) for 5 days. Animals were evaluated twice daily for clinical signs associated with surgery on the lumbar spine such as strong reaction after palpation of the wound, kyphosis, severe ataxia, open anal sphincter, flaccid tail, paresis/paralysis and behavioral signs. The rescue analgesia protocol was consisted of opioid analgesic buprenorphine at a dose of 0.01 mg/kg i/m every 8 h. All animals used in the experiment did not show signs of pain after the surgery and meloxicam was the only analgetic used after the surgical procedures.

Experimental animals were euthanized after 14 (second experiment), 27 (first experiment) and 40 (second experiment) weeks after surgery. Euthanasia was performed by intravenous administration of euthanasia preparations (Euthasol 400 mg/ml) at a dose of 140 mg/kg i.v. following intravenous administration of xylazine (0.1 mg/kg) and ketamine (10 mg/kg).

Approval for the studies was given by the Directorate for Veterinary and Food Safety, Ministry of Agriculture, Republic of Croatia. Ethical principles of the study ensured compliance with European Directive 2010/63/EU, the Law on amendments to the Animal Protection Act (Official Gazette 37/13), the Animal Protection Act (Official Gazette 102/17), the Ordinance on the protection of animals used for scientific purposes (Official Gazette 55/13), ARRIVE guidelines, FELASA recommendations, and recommendations of the Ethics Committee at Faculty of Veterinary Medicine, University of Zagreb and National Ethics Committee (EP 275/2020).

### Radiological evaluation

In both experiments, the lumbar portion of the spine was separated after euthanasia and the success of spinal fusion was preliminarily evaluated using an X-ray. To further analyse the structure and amount of newly formed bone as well as osseointegration with transverse processes, obtained specimens were scanned using a microCT machine (SkyScan 1076, Bruker, Billerica, MA, USA). Before microCT scanning, the spine was sawed into two halves to fit the dimensions of the microCT device. Scanning parameters were as follows: scanning resolution 18 µm, aluminium filter (0.5 mm) and frame averaging set to a value of 2. Acquired data was analysed using CTAn software (Bruker, Billerica, MA, USA) following the reconstruction of images in NRecon software (Bruker, Billerica, MA, USA) as described^2, 3, 13, 55^. In microCT analyses we used global threshold for bone tissue and ceramics to distinguised between them based on the density. Additionally, in the second experiment spinal fusion outcome was evaluated 2 months after implantation using in vivo CT scans (SOMATOM go.Now, Siemens AG, Germany). CT imaging was performed using the following settings: 130 kV, 160 mAs, pitch 1.10 and slice thickness 0.60 mm.

### Manual palpation

Following ex vivo radiological evaluation, the mobility of spine segments was assessed by two independent observers (N.I. and N.S.) as described^13^. In brief, fused transverse processes were compressed with fingers and possible motion between vertebrae was observed. If any motion was absent, fusion was considered successful.

### Bone maceration

The bone maceration technique was conducted according to our standard procedure on one selected specimen from the first experiment to show the gross anatomy of the fused lumbar segment. In brief, the majority of residual muscles and connective tissue were manually removed. Further, the lumbar spine specimen was placed in a metal container containing water with the addition of a mild detergent to facilitate the removal of fat and heated at a constant temperature for several days. Through the process, softened tissue was removed. Finally, the cleaned specimen was left in 10% hydrogen peroxide solution for 5 days to achieve a lighter hue.

### Histology

All spine specimens (except the one used for bone maceration) were processed histologically to analyse the structure of ectopic bone and osseointegration with adjacent transverse processes. Selected samples (three or two per group) were processed undecalcified using the previously described procedure^2, 13, 14^. In brief, following fixation in 10% neutral buffered formalin, an automated tissue processing system (ASP300S, Leica Biosystems, Buffalo Grove, IL, USA) was used to dehydrate samples in graded solutions of ethyl alcohol. Subsequently, samples were polymerized into hardened acrylic resin blocks (MMA). Automated microtome (SM2500, Leica Biosystems, Buffalo Grove, IL, USA) and tungsten-carbide knives (D-profile, Delaware Diamond Knives, Wilmington, DE, USA) were used to obtain 5 µm sections which were mounted on gelatin-coated glass slides. Additionally, selected specimens processed undecalcified were used to obtain ground (35 µm) sections. All remaining specimens were processed decalcified following biomechanical evaluation as previously described^14^. Sections obtained by undecalcified processing were stained with modified Goldner`s trichrome, hematoxylin–eosin (HE), Von Kossa or Sanderson’s Rapid Bone Stain with Van Gieson picrofuchsin while sections obtained following decalcification were stained by Goldner`s trichrome stain.

### Biomechanical testing

Furthermore, to determine the biomechanical properties of induced bone between transverse processes we conducted a three-point bending test on selected specimens (five or four per group) obtained 27 and 40 weeks following implantation. Specimens harvested 14 weeks after surgery were predominantly processed undecalcified and the remaining number of specimens was not sufficient to obtain reliable results. Three-point bending test was conducted using TA.HDplus instrument (Stable Micro Systems, UK) as previously described^2, 13, 14^. In brief, specimens determined for biomechanical testing were dissected to reveal bone between fused transverse processes, placed on two supports, and the force was applied in the midpoint using a single-pronged loading device with the flat-tipped wedge. Three-point bending test determined the maximum force, work to fracture, and deflection of induced bone fused with transverse processes.

### Pathology dissection

At the end of experiments pathology dissection was conducted on all animals by pathology consultants at the Department of Pathology at Faculty of Veterinary Medicine, University of Zagreb. Pathology dissection was conducted according to the standard operating procedure of the Department of Pathology. In brief, each animal was dissected and following thorough examination samples of vital organs (heart, lungs, brain, liver, spleen, kidneys, stomach, intestine, skeletal muscle) were harvested and analysed after histological processing.

### Data management

To check does the data follows Gaussian or non–Gaussian distribution Kolmogorov–Smirnov test was employed. Gaussian–distributed data was analysed using an unpaired t-test (two experimental groups) or two-way ANOVA with Tukey's multiple comparisons tests (three or more experimental groups). Non–Gaussian distributed data was analysed with the Mann – Whitney U test (two experimental groups) or the Kruskal–Wallis test with Dunn's multiple comparisons test (three or more experimental groups). Data are shown as mean with standard deviation (SD) or as median with minimum and maximum. Significant P (P < 0.05) values are marked with with asterisks; *(P ≤ 0.05), **(P ≤ 0.01), ***(P ≤ 0.001). GraphPad Prism software (v.8.4.3) was used in all statistical analyses.

### Supplementary Information

Supplementary Information.

## Data Availability

Raw data were generated at the Laboratory for Mineralized Tissues. Derived data supporting the findings of this study are available from the corresponding author, S.V., upon request.
