# Supplementary material for: Calcium phosphate ceramics combined with rhBMP6 within autologous blood coagulum promote posterolateral lumbar fusion in sheep
Source: Sci Rep. 2023 Dec 12;13:22079. doi: 10.1038/s41598-023-48878-9 (PMC10716416; doi:10.1038/s41598-023-48878-9)
Supplement: Supplementary file 1 — Supplementary Information. [file 41598_2023_48878_MOESM1_ESM.docx]

**STUDY LIMITATIONS**

The limitations of the present study include a relatively small number of specimens used in biomechanical testing because a certain number of specimens were histologically processed to present the entire fusion mass including the adjacent transverse processes. Moreover, the applied biomechanical method (three-point bending test) evaluated specimens in only one loading direction, and thus the biomechanical testing was consequently not conducted in all spine loading directions.
